# Contributions from Patients

**Published:** 1920-04-03

**Authors:** 


### CONTRIBUTIONS FROM PATIENTS.

There is much of interest in the various views expressed in The Hospital by different managers upon the proposal to make a weekly charge of 10s. from each in-patient towards the cost of his food. Is the principle generally favoured? Let us collate the opinions which we have already published. There can be no question that opinion is divided and that this division does not entirely depend upon the circumstances of the different institutions. We believe that it would be fair to say that if contributions from patients could be avoided almost everyone would be pleased; at least it is so in London.

Generally speaking, the special hospitals have been to the fore in this matter; the children's hospitals are inclined to think the proposal impracticable for themselves; and the opinion of the general hospitals seems to depend upon the area in which they are situated. Those which depend upon weekly contributions from workmen (though there are exceptions to this rule), the hospitals in industrial areas, fear that since the men contribute while they are well they would take it ill to be asked to contribute when they are sick. Those who have not already tried the two systems believe that they are hostile to each other. In the main the plan of contributions from patients is regarded more favourably in London than in the provinces. It is also plain that many would prefer a graduated system of free wards and pay wards on the American plan to a payment required from an ordinary patient. Indeed the distinction between food and treatment is largely fictitious; and it would be preferable to ask for a contribution towards the present cost of maintenance than to let it be regarded as in any sense a payment towards any item in the general bill. Beside this, the system of a flat rate has many critics. Those, chiefly from the special hospitals who have experience of the plan, declare that one sum is as easy to collect as another; that inquiries have always to be made to avoid hardships; that ten shillings is sometimes too large and sometimes too small; and that ability and willingness to pay should decide the amount which might vary from half-a-crown to a gift of three guineas.

With one or two exceptions, the provincial hospitals which do not favour contributions from patients express a lively faith in their power to increase their incomes by developing their traditional sources of revenue. We would commend to our readers the action of the Mayor of Newport, who believes that he can raise £200,000 for the Royal Gwent Hospital, and has started the plan described in one of our notes. His confidence, which is shared by many men in the provinces, is an exhilarating contrast to the pessimistic fog in London. Yet, if £200,000 can be raised without a miracle in Newport, a million should be capable of being raised without a miracle in the City of London alone. And the fact remains that the voluntary system in the past has been supported by contributions from the well, not by contributions from the sick, and that the most enthusiastic supporters of the new charge do not hope that it will do more than somewhat lessen their difficulties.

Consequently, the importance of the proposal should not be exaggerated. We must remember that existing sources of income must be developed. How is this to be done? The movement is unpropitious for the issue of further appeals. But that fact is not at all discouraging. What the Royal Victoria Hospital in Belfast has accomplished by private visits to the head of the firms in *every* trade, might surely be accomplished in London. Have the firms in the City, for instance, been regularly approached in this way for annual subscriptions? We do not believe they have. The total sum required by the hospitals in London is so small compared to the wealth in the City that it can be only the method which is at fault if the money required is not raised.

